# DNA methylation profiling of the X chromosome reveals an aberrant demethylation on CXCR3 promoter in primary biliary cirrhosis

**DOI:** 10.1186/s13148-015-0098-9

**Published:** 2015-07-07

**Authors:** Ana Lleo, Weici Zhang, Ming Zhao, Yixin Tan, Francesca Bernuzzi, Bochen Zhu, Qian Liu, Qiqun Tan, Federica Malinverno, Luca Valenti, Tingting Jiang, Lina Tan, Wei Liao, Ross Coppel, Pietro Invernizzi, Qianjin Lu, David H. Adams, M. Eric Gershwin, the PBC Epigenetic Study Group

**Affiliations:** Liver Unit and Center for Autoimmune Liver Diseases, Humanitas Clinical and Research Center, Rozzano, Milan Italy; Department of Dermatology, Hunan Key Laboratory of Medical Epigenomics, The Second Xiangya Hospital, Central South University, Changsha, Hunan People’s Republic of China; Migliavacca Center for the Study of Liver Disease, 1st Division of Gastroenterology, Fondazione Istituto di Ricovero e Cura a Carattere Scientifico (IRCCS) Ca’ Granda - Ospedale Maggiore Policlinico Milano, Milan, Italy; Internal Medicine, Fondazione Istituto di Ricovero e Cura a Carattere Scientifico (IRCCS) Ca’ Granda - Ospedale Maggiore Policlinico Milano, Milan, Italy; Department of Microbiology, Monash University, Clayton, Victoria Australia; Division of Rheumatology, Allergy and Clinical Immunology, University of California at Davis School of Medicine, Genome and Biomedical Sciences Facility, 451 Health Sciences Drive, Suite 6510, Davis, CA 95616 USA; Centre for Liver Research and NIHR Biomedical Research Unit for Liver Disease, University of Birmingham, Edgbaston, Birmingham, England UK

**Keywords:** X chromosome, Primary biliary cirrhosis, CXCR3

## Abstract

**Background:**

Although the etiology of primary biliary cirrhosis (PBC) remains enigmatic, there are several pieces of data supporting the thesis that a strong genetic predisposition and environmental factors interact to produce a selective loss of tolerance. The striking female predominance of PBC has suggested that this sex predisposition may be secondary to epigenetic alterations on the X chromosome. In the present study, we rigorously defined the X chromosome methylation profile of CD4, CD8, and CD14 cells from 30 PBC patients and 30 controls. Genomic DNA from sorted CD4, CD8, and CD14 subpopulations was isolated, sonicated, and immunoprecipitated for analysis of methylation. All products were hybridized to a custom-tiled four-plex array containing 27,728 CpG islands annotated by UCSC and 22,532 well-characterized RefSeq promoter regions. Furthermore, bisulfite sequencing was then used for validation on a subsequent group of independent samples from PBC patients and controls. Thence, expression levels of selected X-linked genes were evaluated by quantitative real-time PCR with cDNA samples from all subjects.

**Results:**

We report herein that a total of 20, 15, and 19 distinct gene promoters reflected a significant difference in DNA methylation in CD4+ T, CD8+ T, and CD14+ cells in patients with PBC. Interestingly, there was hypermethylation of FUNDC2 in CD8+ T cells and a striking demethylation of CXCR3 in CD4+ T cells, which inversely correlated with CXCR3 expression levels in CD4+ T cells from early-stage PBC patients.

**Conclusions:**

Our data provides a set of genes with epigenetic alteration likely to be indicators of autoimmunity and emphasizes the role of CXCR3 in the natural history of PBC.

**Electronic supplementary material:**

The online version of this article (doi:10.1186/s13148-015-0098-9) contains supplementary material, which is available to authorized users.

## Background

Autoimmune diseases are multifactorial in origin. Genetic background confers susceptibility to disease onset but is not sufficient for disease development. Epigenetic mechanisms such as DNA methylation and histone modifications regulate gene expression levels and provide an alternative mechanism for modulating gene function and genetic changes. Importantly, DNA methylation plays an important role in T cell differentiation [1, 2], and the potential role of epigenetics in environment and genetics interaction has been widely suggested in autoimmunity [3, 4]. With these data in mind, we note that primary biliary cirrhosis (PBC) is characterized by the presence of high titers of circulating antimitochondrial antibodies (AMAs), innate immune system activation [5–7], and liver-infiltrating autoreactive CD4 and CD8 T lymphocytes [8–10], leading to the progressive destruction of small intrahepatic bile ducts [11, 12]. Genetic polymorphisms account for individual susceptibility to PBC in a minority of cases, as supported by genome-wide association (GWAS) data [13–17], while environmental factors may act through epigenetic changes, as demonstrated for specific genes [18–20]. It has long been known that there is a striking female sex bias in PBC [21], and recent data has suggested involvement of sex chromosomes [22, 23]. For example, an enhanced X monosomy rate in peripheral lymphocytes has been demonstrated in women with PBC [24], despite random X chromosome inactivation [25], as well as an increased Y loss in men with PBC [26].

We thus hypothesized that there is an X chromosome epigenetic component in PBC. Herein we performed an extensive X chromosome methylation analysis in CD4, CD8, and CD14 cells from PBC patients and controls, coupled with gene expression profile from the same cells. The frequency of CXCR3-expressing cells in peripheral blood is known to be higher in PBC patients, and CXCR3-positive cells are found in the portal areas of diseased livers, primarily on CD4+ T cells [27]. Moreover, we report that the CXCR3 promoter is demethylated in CD4+ T cells from PBC patients and the expression level of CXCR3 in CD4+ T cells is significantly increased in comparison to controls and inversely correlated with the overall methylation levels of the CXCR3 promoter, indicating that promoter demethylation status contributed to CXCR3 overexpression in CD4+ T cells. Hence, we submit that there is an epigenetic mechanism that underlies CXCR3 expression in PBC. Our findings not only identify potentially relevant DNA methylation markers for the clinical characterization of PBC patients but also support the thesis that epigenetic changes may be critical for the clinical manifestations of PBC.

## Results

### DNA methylation

**Fig. 1:**
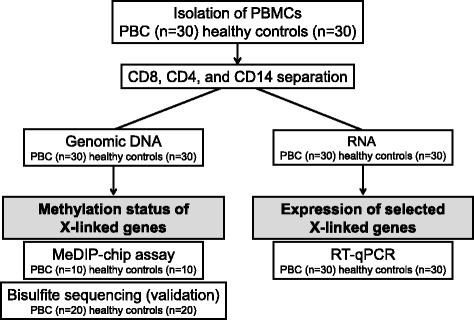
Flow chart illustrating the study design

**Fig. 2:**
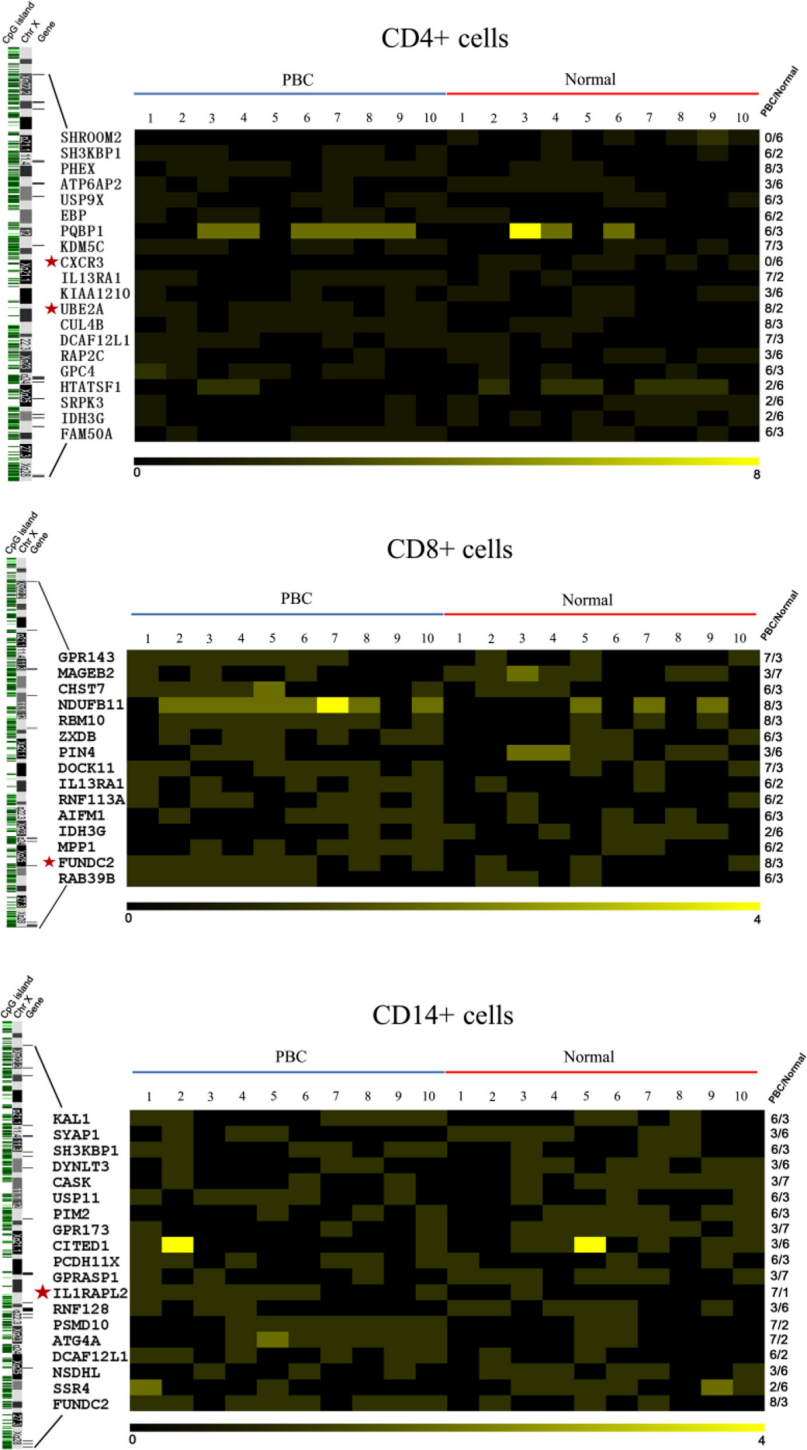
Differential methylation between PBC-affected subjects (*n* = 10) and healthy controls (*n* = 10) in CD4+, CD8+, and CD14+ cells represented in a heat map. The genomic locations of genes are shown on the *left* of the X chromosome ideogram. On the *right*, different methylated regions (DMR) of genes are identified in at least 6/10 samples in one group and 3/10 samples in the other. The predicted DMR along with the names of the genes in each cell examined is depicted as a two-dimensional heat map, respectively. *Black blocks* represent zero peaks, and *yellow blocks* symbolize different peak numbers corresponding to the *scale bar* at the bottom

**Fig. 3:**
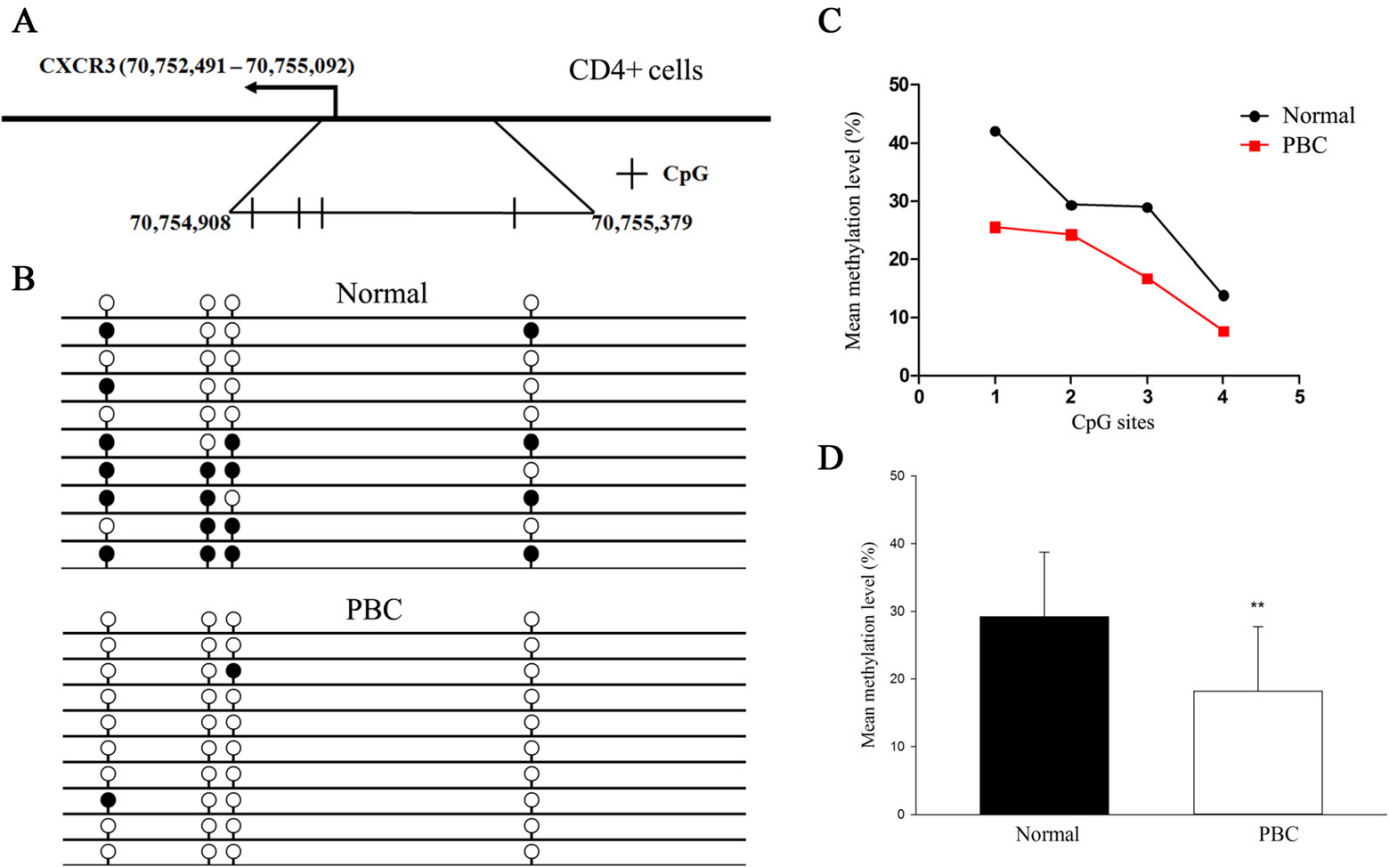
Bisulfite sequencing-based DNA methylation analysis of the CXCR3 promoter region in CD4+ T cells. **a** Chromosomal sequence location of the CXCR3 promoter that was analyzed by bisulfite sequencing. This region contains four CpG sites (*vertical bars*) within a 471 bp region overlapping the transcription start site as shown. **b** Representative bisulfite sequencing data from CD4+ cells of a PBC sample and a normal control, respectively. Each *circle* represents a CpG site, and each *line* represents an individually sequenced clone. *Filled circles* represent methylated sites (protected from bisulfite conversion), while *unfilled circles* represent unmethylated CpG sites (converted). **c** Mean methylation level was calculated for each individual CpG site within the CXCR3 promoter region for all clones for PBC (*n* = 20) versus healthy (*n* = 20) CD4+ cells. **d** Overall percentage of methylation of CXCR3 was determined by the total number of methylated sites out of the total CpG sites in all clones and graphed as mean ± SD for PBC (*n* = 20) versus healthy (*n* = 20) CD4+ cells. **p < 0.01

**Fig. 4:**
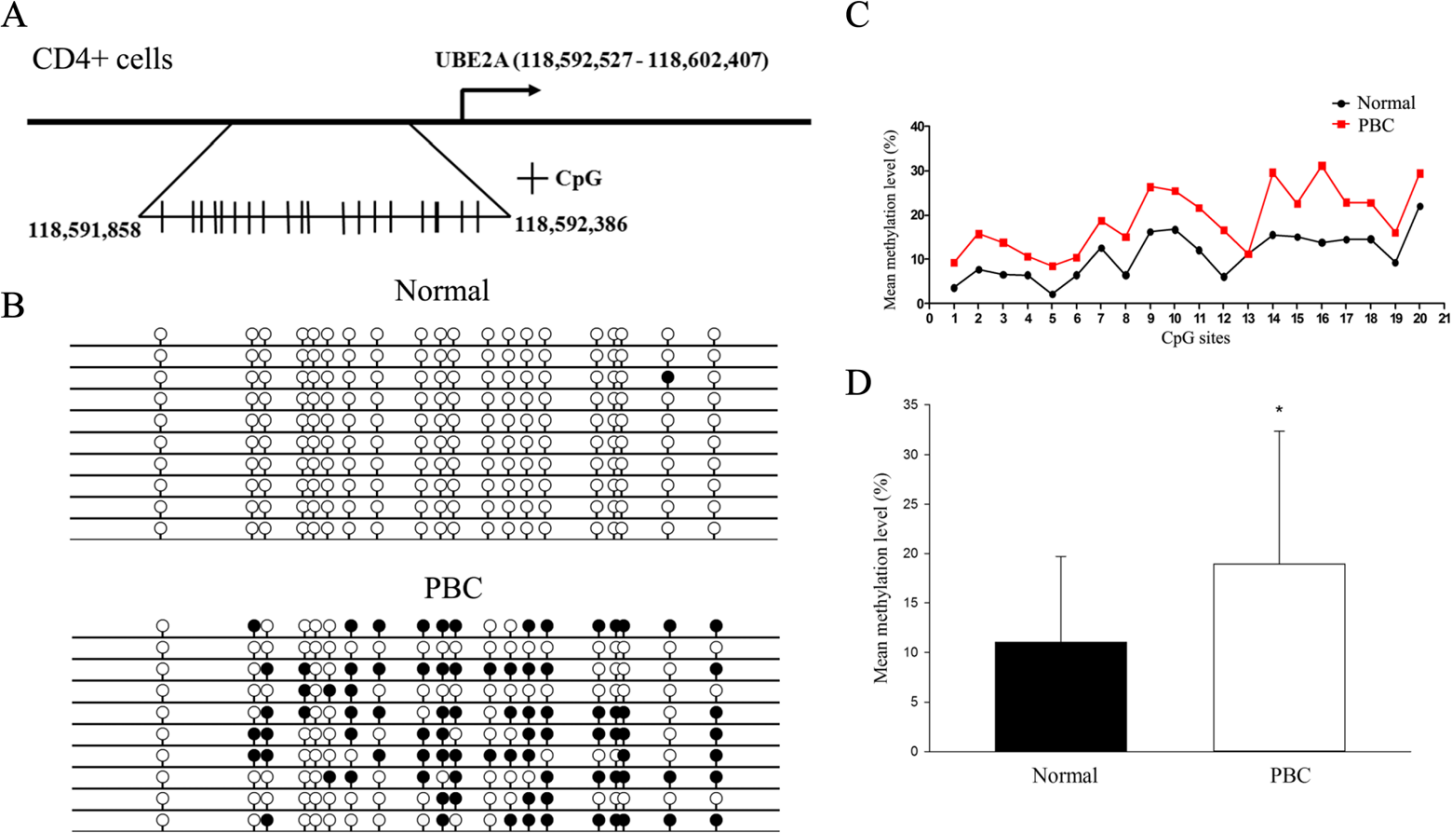
**a** Chromosomal sequence location of the region of the UBE2A promoter that was analyzed by bisulfite sequencing. This region contains 20 CpG sites (*vertical bars*) within a 542 bp region upstream of the transcription start site as shown. **b** Representative bisulfite sequencing data from CD4+ cells of a PBC sample and a normal control, respectively. Each *circle* represents a CpG site, and each *line* represents an individually sequenced clone. *Filled circles* represent methylated sites (protected from bisulfite conversion), while *unfilled circles* represent unmethylated CpG sites (converted). **c** Mean methylation level was calculated for each individual CpG site within the UBE2A promoter region for all clones for PBC (*n* = 20) versus healthy (*n* = 20) CD4+ cells. **d** Overall percentage of methylation of UBE2A was determined by the total number of methylated sites out of the total CpG sites in all clones and graphed as mean ± SD for PBC (*n* = 20) versus healthy (*n* = 20) CD4+ cells. *p < 0.05

**Fig. 5:**
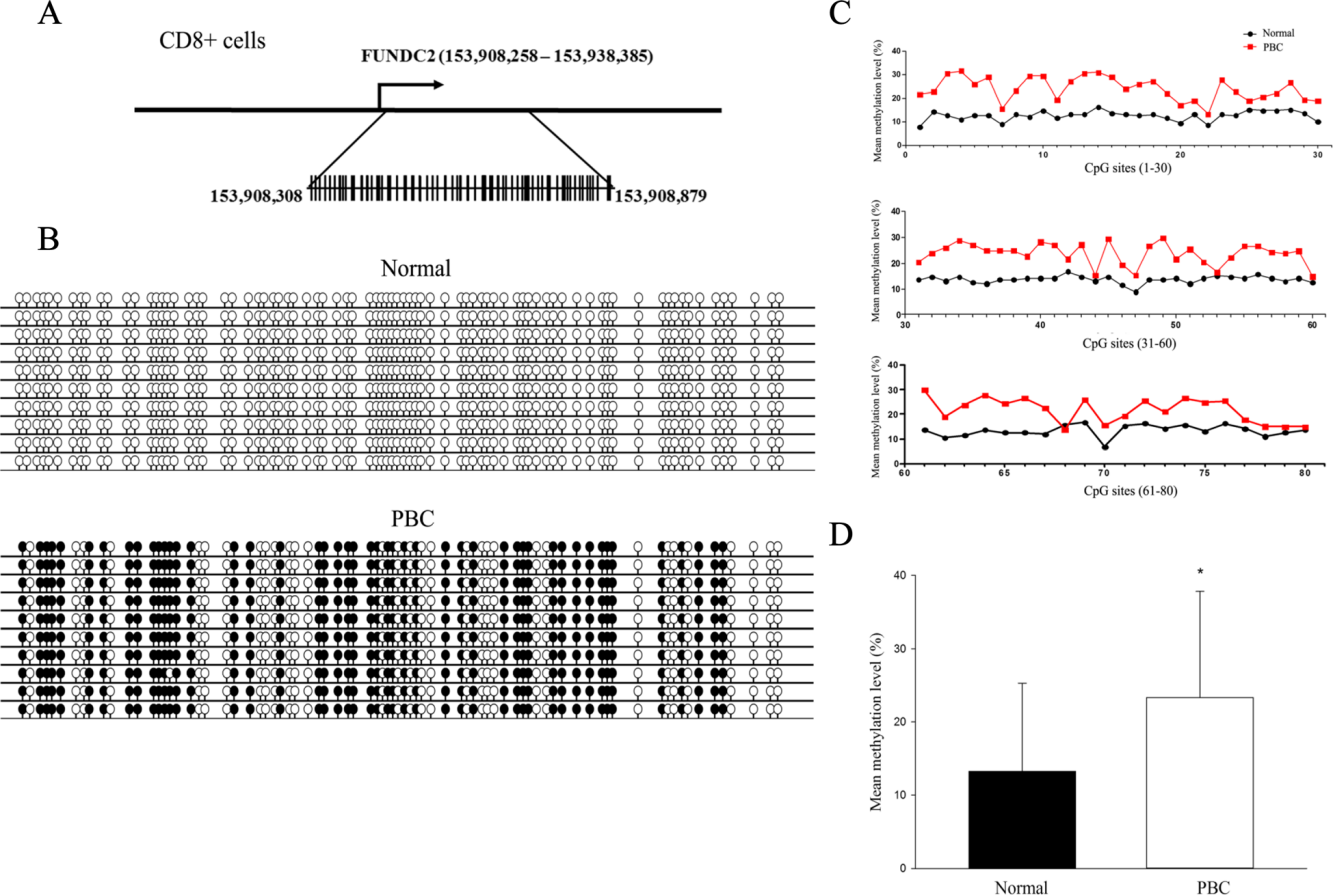
**a** Chromosomal sequence location of the region of the FUNDC2 promoter that was analyzed by bisulfite sequencing. This region contains 80 CpG sites (*vertical bars*) within a 696 bp region close to the transcription start site as shown. **b** Representative bisulfite sequencing data from CD8+ cells of a PBC sample and a normal control, respectively. Each *circle* represents a CpG site, and each *line* represents an individually sequenced clone. *Filled circles* represent methylated sites (protected from bisulfite conversion), while *unfilled circles* represent unmethylated CpG sites (converted). **c** Mean methylation level was calculated for each individual CpG site within the FUNDC2 promoter region for all clones for PBC (*n* = 20) versus healthy (*n* = 20) CD8+ cells. **d** Overall percentage of methylation of FUNDC2 was determined by the total number of methylated sites out of the total CpG sites in all clones and graphed as mean ± SD for PBC (*n* = 20) versus healthy (*n* = 20) CD8+ cells. *p < 0.05

**Fig. 6:**
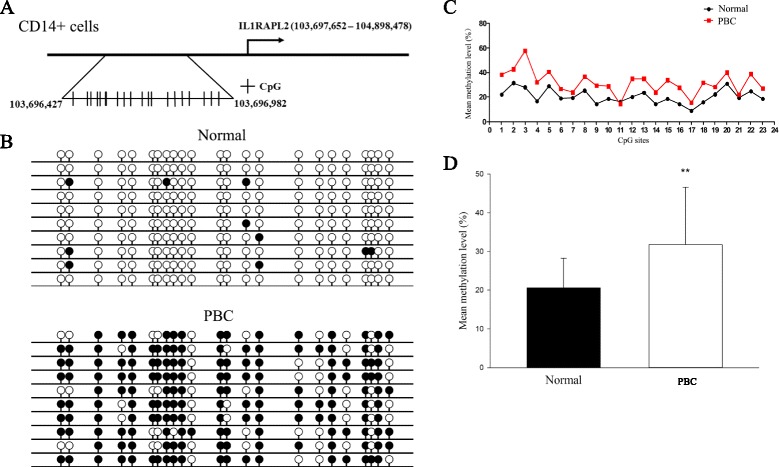
**a** Chromosomal sequence location of the region of the IL1RAPL2 promoter that was analyzed by bisulfite sequencing. This region contains 23 CpG sites (*vertical bars*) within a 423 bp region upstream of the transcription start site as shown. **b** Representative bisulfite sequencing data from CD14+ cells of a PBC sample and a normal control, respectively. Each *circle* represents a CpG site, and each *line* represents an individually sequenced clone. *Filled circles* represent methylated sites (protected from bisulfite conversion), while *unfilled circles* represent unmethylated CpG sites (converted). **c** Mean methylation level was calculated for each individual CpG site within the IL1RAPL2 promoter region for all clones for PBC (*n* = 20) versus healthy (*n* = 20) CD14+ cells. **d** Overall percentage of methylation of IL1RAPL2 was determined by the total number of methylated sites out of the total CpG sites in all clones and graphed as mean ± SD for PBC (*n* = 20) versus healthy (*n* = 20) CD14+ cells. **p < 0.01

Initially, X chromosome methylated DNA immunoprecipitation (MeDIP) arrays were performed on cell subsets from 10 PBC patients and 10 controls (Fig. 1). We identified 8 hypomethylated genes and 12 hypermethylated genes in CD4+ cells, 3 hypomethylated genes and 12 hypermethylated genes in CD8+ cells, and 9 hypomethylated genes and 10 hypermethylated genes in CD14+ cells in PBC compared with normal controls (Fig. 2, Table 1). Gene ontology (GO) analysis was conducted to identify the cell component, molecular function, and biological processes of these genes. Bisulfite sequencing was thence used to validate the results of the MeDIP-ChIP. Based on the GO analysis, four genes were chosen for validation in CD4+, CD8+, and CD14+ cells from 20 additional PBC patients and 20 additional normal controls. Importantly, CXCR3 was demonstrated to be significantly hypomethylated in CD4+ T cells from PBC patients (Fig. 3), whereas UBE2A and FUNDC2 were hypermethylated in CD4+ T cells and CD8+ T cells, respectively, in PBC patients (Figs. 4 and 5). IL1RAPL2 was hypermethylated in CD14+ cells in PBC (Fig. 6).

**Table 1:** Differentially methylated genes between PBC patients (n = 10) and controls (n = 10) in CD4+ T cells, CD8+ T cells, and CD14+ cells from MeDIP array

Gene symbol	Promoter methylation	Chromosome location	Peak start	Peak end	Molecular function
CD4+ T cells					
SHROOM2	Hypo	Xp22.3	9712072	9712217	Protein binding; actin binding; ion channel activity
ATP6AP2	Hypo	Xp11.4	40325091	40325230	Receptor activity; protein binding; peptidase activity
CXCR3	Hypo	Xq13	70754908	70755379	Protein binding; receptor activity
KIAA1210	Hypo	Xq24	118168783	118168934	–
RAP2C	Hypo	Xq25	131181458	131182027	Nucleotide binding; hydrolase activity
HTATSF1	Hypo	Xq26.3	135407214	135407962	RNA binding; nucleotide binding
SRPK3	Hypo	Xq28	152699372	152700237	Protein binding; nucleotide binding; protein kinase activity
IDH3G	Hypo	Xq28	152712248	152713105	Nucleotide binding; catalytic activity; binding
SH3KBP1	Hyper	Xp22.1-p21.3	19815048	19815499	Kinase activity; protein binding
PHEX	Hyper	Xp22.2-p22.1	21960765	21961408	Binding; peptidase activity
USP9X	Hyper	Xp11.4	40829716	40830189	–
EBP	Hyper	Xp11.23-p11.22	48265283	48265716	–
PQBP1	Hyper	Xp11.23	48638698	48639426	DNA binding; protein binding; transcription regulator activity
KDM5C	Hyper	Xp11.22-p11.21	53270826	53271657	Catalytic activity; binding; DNA binding; transferase activity
IL13RA1	Hyper	Xq24	117745388	117746245	Protein binding; receptor activity
UBE2A	Hyper	Xq24	118592639	118593104	Catalytic activity; protein binding; nucleotide binding
CUL4B	Hyper	Xq23	119578240	119579025	Protein binding
DCAF12L1	Hyper	Xq25	125513992	125514963	–
GPC4	Hyper	Xq26.1	132376771	132377010	Protein binding
FAM50A	Hyper	Xq28	153325585	153326034	–
CD8+ T cells					
MAGEB2	Hypo	Xp21.3	30142775	30143514	–
PIN4	Hypo	Xq13	71318699	71318834	DNA binding; catalytic activity
IDH3G	Hypo	Xq28	152713172	152713419	Nucleotide binding; catalytic activity; binding
GPR143	Hyper	Xp22.3	9693469	9693810	Binding; protein binding
CHST7	Hyper	Xp11.23	46317790	46318126	Transferase activity
NDUFB11	Hyper	Xp11.23	46889596	46889747	–
RBM10	Hyper	Xp11.23	46889596	46889747	RNA binding; nucleotide binding
ZXDB	Hyper	Xp11.21	57635489	57636052	Nucleic acid binding; binding; protein binding; sequence-specific DNA binding transcription factor activity
DOCK11	Hyper	Xq24	117514077	117514418	Enzyme regulator activity; lipid binding; protein binding
IL13RA1	Hyper	Xq24	117745582	117746125	Protein binding; receptor activity
RNF113A	Hyper	Xq24	118889675	118890227	Binding; protein binding
AIFM1	Hyper	Xq26.1	129101688	129102158	Catalytic activity; nucleotide binding; protein binding; electron carrier activity; DNA binding
MPP1	Hyper	Xq28	153686370	153686806	Kinase activity; protein binding
FUNDC2	Hyper	Xq28	153908428	153908659	–
RAB39B	Hyper	Xq28	154146437	154146790	Nucleotide binding
CD14+ cells					
SYAP1	Hypo	Xp22.2	16647938	16648203	–
DYNLT3	Hypo	Xp21	37591227	37591696	–
CASK	Hypo	Xp11.4	41667126	41667877	Protein binding; ATP binding; protein kinase activity
GPR173	Hypo	Xp11	53095323	53095568	G protein-coupled receptor activity
CITED1	Hypo	Xq13.1	71441965	71442118	Sequence-specific DNA binding transcription factor activity; protein binding; DNA binding; transcription regulator activity; chromatin binding; hydrolase activity
GPRASP1	Hypo	Xq22.1	101790525	101790970	Protein binding
RNF128	Hypo	Xq22.3	105824083	105824257	Binding; catalytic activity
NSDHL	Hypo	Xq28	151750127	151750280	Catalytic activity
SSR4	Hypo	Xq28	152710848	152711207	Calcium ion binding
KAL1	Hyper	Xp22.32	8659278	8659655	Enzyme regulator activity; protein binding; carbohydrate binding; structural molecule activity
SH3KBP1	Hyper	Xp22.1-p21.3	19815148	19815397	Protein binding
USP11	Hyper	Xp11.23	46976944	46977091	Peptidase activity; protein binding; hydrolase activity
PIM2	Hyper	Xp11.23	48661207	48661380	Protein kinase activity; nucleotide binding
PCDH11X	Hyper	Xq21.3	90921374	90921541	Calcium ion binding
IL1RAPL2	Hyper	Xq22	103696629	103696884	Protein binding; receptor activity
PSMD10	Hyper	Xq22.3	107221347	107221874	Protein binding
ATG4A	Hyper	Xq22.1-q22.3	107221347	107221874	Peptidase activity
DCAF12L1	Hyper	Xq25	125513614	125513941	Protein binding
FUNDC2	Hyper	Xq28	153908232	153908879	–

### Gene expression

**Fig. 7:**
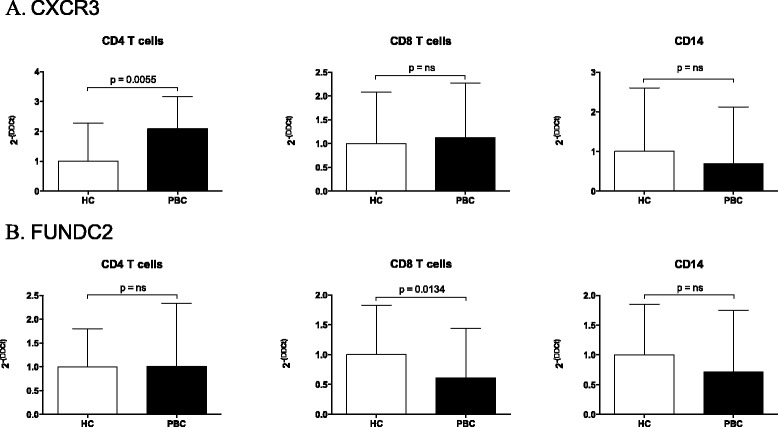
CXCR3 (**a**), and FUNDC2 (**b**) expression level was determined in CD4+, CD8+ T cells, and CD14+ cells in PBC patients and controls. Real-time threshold cycle values for cDNA were normalized with GADPH and compared to a calibrator. Limits (95 % CI) of the best-fit line are shown

CXCR3, UBE2A, FUNDC2, and IL1RAPL2 expression was studied by reverse transcription and quantitative polymerase chain reaction (RT-qPCR) in CD4+, CD8+, and CD14+ cells in a cohort of 30 PBC and 30 normal controls (Fig. 7).

**Fig. 8:**
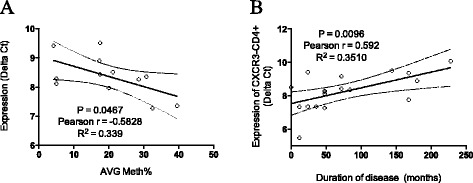
Correlation analysis was performed to evaluate the relationship between CXCR3 gene expression levels, methylation status of CXCR3 promoter, and disease duration. **a** CXCR3 expression in CD4+ T cells inversely correlates with methylation status of CXCR3 promoter. **b** CXCR3 expression in CD4+ T cells correlates with disease duration. Real-time threshold cycle values for CXCR3 cDNA were normalized with GADPH and compared to a calibrator. Calibrated CXCR3 expression levels were correlated to disease duration (months). 95 % confidence limits of the best-fit line are shown (*solid line*)

Importantly, CXCR3 was significantly overexpressed in CD4+ T cells in PBC (*p* = 0.0055), whereas FUNDC2 was significantly less expressed in CD8+ T cells from PBC than controls (*p* = 0.0134). These results are concordant with the methylation data. IL1RAPL2 was not expressed in any cell subset in PBC or controls, and no significant differences were identified for UBE2A in the three subsets investigated (data not shown). Finally, a significant negative correlation between CXCR3 expression and overall methylation levels in CD4+ T cells was found in early-stage PBC patients (*p* < 0.05) (Fig. 8a). Of note, there was no difference observed based on UDCA treatment (data not shown). In addition, our data demonstrated that CXCR3 expression levels in CD4+ T cells were significantly associated with duration of disease (*p* < 0.01) (Fig. 8b) but not associated with age (data not shown).

### CD4 T cell phenotype

**Fig. 9:**
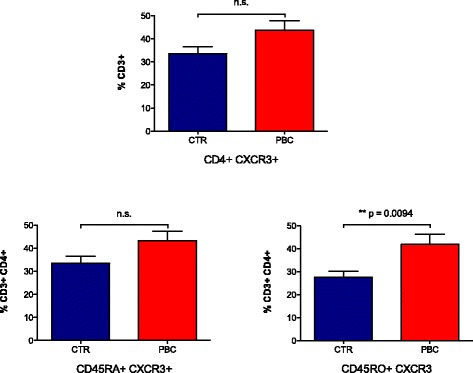
CD4 T cell populations expressing CXCR3 in PBC (*n* = 20) and controls (*n* = 10) were quantified by flow cytometry. In PBC, there is an increased number of CD4 T cells in the periphery expressing CXCR3, in both naïve (CD45RA) and memory cells (CD45RO). Activated CD4+CD45RO+ cells express significantly higher levels of CXCR3 in PBC (41.82 % ± 4.37) compared to controls (27.57 % ± 2.61) (*p* = 0.0094)

Finally, we quantified CD4 T cell populations expressing CXCR3 in both PBC (*n* = 20) and controls (*n* = 10) by flow cytometry. Based on the expression data, patients with PBC had an increased number in the periphery of CD4 T cells expressing CXCR3. In addition, both naïve and memory CD4 T cells from PBC expressed a higher frequency of CXCR3 cells in the periphery than controls. However, only activated CD4+CD45RO+ cells expressed significantly higher levels of CXCR3 (41.82 % ± 4.37 versus 27.57 % ± 2.61, *p* = 0.0094) (Fig. 9). Please note we included both early (*n* = 10) and advanced (*n* = 10) stage patients with PBC (data not shown).

## Discussion

As the case for most autoimmune diseases, PBC results from the contribution of genetic and environmental factors, reflected by incomplete concordance in monozygotic twins [28]. Moreover, our group has suggested a significant role for X chromosome defects in PBC, based on the observation that women with PBC have a significantly enhanced monosomy X frequency in peripheral white blood cells compared to age- and disease stage-matched women with chronic viral hepatitis and to age-matched healthy women [24]. Epigenetic mechanisms act at the interface of genetic and environmental influences on human phenotype and disease risk by altering gene expression levels without altering DNA sequence or chromosome structure. In the case of autoimmune diseases, epigenetic mechanisms have been implicated in the pathogenesis of systemic lupus erythematosus [29, 30] and type I diabetes [31]. Moreover, we had previously explored the X chromosome epigenetic component in PBC [18].

Importantly, PBC is a complex autoimmune disease involving autoreactive CD4+ and CD8+ T cell responses, present in human circulating lymphocytes and liver, B cell compartment, and innate immunity, including macrophages [6, 32–34].

In this study, we investigated for the first time the DNA methylation status of the X chromosome in CD4+, CD8+, and CD14+ cells isolated from PBC patients and well-matched controls. We herein demonstrate that the CXCR3 promoter is demethylated in CD4+ T cells from PBC patients compared to controls, which is supported by a significantly higher expression of CXCR3 in the same cell subtype of PBC patients. CXCR3 gene encodes a G protein-coupled receptor with selectivity for three chemokines: CXCL9 or MIG (monokine induced by IFNγ), CXCL10 or IP10 (IFNγ-inducible 10 kDa protein), and CXCL11 or I-TAC (IFN-inducible T cell α-chemoattractant). CXCR3 plays an important role in regulating leukocyte trafficking; indeed binding of chemokines induces cellular responses that are involved in leukocyte traffic, most notably integrin activation, cytoskeletal changes, and chemotactic migration. CXCR3 is expressed primarily on activated T lymphocytes and NK cells and some epithelial cells and endothelial cells. It had been implicated that CXCR3 is highly expressed on pro-inflammatory human CD4+Th17 cells [35], “pathogenic” human Th1/17 cells [36], and myelin oligodendrocyte glycoprotein-specific CD4+ T cells in experimental autoimmune encephalomyelitis mice in combination with other biomarkers [37]. Increased CXCR3 in portal tracts has also been reported in PBC patients [27]. Additionally, levels of CXCR3 ligands are markedly increased in inflammatory liver diseases [38, 39], including PBC [27]. Further, knockout of CXCR3 delays PBC progression in an animal model [40]. Most importantly, our data indicates that CXCR3 expression in CD4+ T cells inversely correlates with methylation levels and positively correlates with duration of disease. The accumulated data suggest a critical role of the CXCR3/chemokine system in the development of autoimmune disorders. In addition to its role in lymphocyte recruitment to tissues, CXCR3 is also implicated in the development of Th1 cells within lymph nodes [41]; thus, hypomethylation of CXCR3 and the consequent increased expression on CD4 T cells could promote disease progression through enhanced Th1 differentiation and increased recruitment. X chromosome demethylation has been shown to result in increased CXCR3 expression in lupus, suggesting this could be a common mechanism that contributes to the female susceptibility to autoimmune diseases [4]. Our findings are in agreement with previous studies; nevertheless, our findings strongly suggest an epigenetic mechanism underlying the elevated CXCR3 expression in PBC.

There are significant limitations in our study that should be discussed. First, of the genes studied herein, we note there were no significant differences identified for UBE2A gene expression, although UBE2A has been demonstrated to be hypermethylated in CD4+ T cells. This suggests that UBE2A expression is regulated by other epigenetic mechanisms rather than by methylation. Second, the pathogenetic mechanisms that underlie CXCR3 demethylation remain to be defined. Third, our study did not include men with PBC. This is of particular interest given the relevant function of the X chromosome. Fourth, the CD4 bias is interesting and it will be important to determine if this bias is found on other T cell subpopulations, particularly Th17, Tregs, or Th1 cells, all of which require CXCR3 to enter the liver. Finally, further studies will also need to focus on the differential expression of central versus effector memory cells. Our data highlight, however, the critical importance of epigenetics in PBC and likely for other autoimmune diseases.

## Conclusions

CXCR3 promoter demethylation in CD4+ T cells from PBC patients, supported by a significantly higher expression of CXCR3 in the same cell subtype, emphasizes the well-known role of CXCR3 in the pathogenesis of PBC. Moreover, CXCR3 expression levels in CD4+ T cells are significantly associated with duration of disease. Whether CXCR3 may contribute to the female susceptibility to PBC warrants further investigation.

## Methods

### Subjects

Fresh heparinized peripheral blood samples were obtained from 30 patients diagnosed with PBC and 30 unaffected age- and sex-matched controls [11]. All patients (Table 2) were female, and 90 % had readily detectable AMA; the diagnosis was made based on internationally accepted criteria [11]. The mean age was 61 (43–78) years old (range years), and 100 % of them were taking ursodiol. Four percent of the PBC patients included in this study were histologically characterized as cirrhotics. Serum liver function tests were performed utilizing routine laboratory methods. Subjects were excluded from the study if they had malignancies or were using immunosuppressive drugs. Patients and controls were matched for age and sex. After approval from appropriate institutional review board, all subjects provided written informed consent prior to enrollment in the study. The study design is illustrated in Fig. 1.

**Table 2:** Clinical, biochemical, and serological characteristics of PBC patients

	PBC patients	Healthy controls
	(*n =* 30)	(*n =* 30)
Mean age (years, range)	64 (44–87)	60 (42–79)
Females (*n*, %)	30 (100 %)	30 (100 %)
AMA positivity (*n*, %)	28 (93 %)	n.a.
Liver cirrhosis (*n*, %)	4 (13 %)	n.a.
Total bilirubin (mg/dl) (n.v. <1.0)	0.64 ± 0.39	0.73 ± 0.18
Alkaline phosphatase (IU/l) (n.v. <279)	152.3 ± 207.3	168 ± 24
Alanine aminotransferase (U/l) (n.v. <50)	33 ± 26	32 ± 9
Albumin (g/dl) (n.v. >3.5)	4.33 ± 0.86	4.68 ± 0.03
Length of follow-up (months, range)	34 (14–56)	n.a.
Therapy with UDCA (*n*, %)	28 (93 %)	n.a.
Liver biopsy (*n*, %)	21 (72 %)	n.a.

Mean values ± standard deviation unless otherwise stated

*n.a.* not applicable, *n.v.* normal value

### CD14+, CD4+ T cell, and CD8+ T cell purification

Peripheral blood mononuclear cells (PBMC) were isolated by centrifugation on a Ficoll-Hypaque gradient for 30 min at 500*g*. First, CD14+ cells were isolated from PBMC by positive selection under endotoxin-free conditions using anti-CD14 conjugated microbeads (Miltenyi Biotec, San Diego, CA, USA). Thence, CD8+ cells were isolated from the eluate by positive selection using anti-CD8 conjugated microbeads (Miltenyi Biotec, San Diego, CA, USA). Finally, the CD4+ T cells were isolated by negative selection using a cocktail of antibodies against CD8, CD14, CD16, CD19, CD36, CD56, CD123, TCR γ/δ, and CD235a (Miltenyi Biotec, San Diego, CA, USA). Aliquots of the CD14+, CD8+, and CD4+ T cells were subjected to viability assays and flow cytometry analysis. The purity of these lymphocytic populations was >95 % and the viability >95 %. Genomic DNA from the isolated CD14+, CD8+, and CD4+ T cells was isolated using TRI Reagent (Sigma-Aldrich, St. Louis, MO, USA) [42].

### Methylated DNA immunoprecipitation and methylation microarrays

DNA samples from PBC patients (*n* = 10) and controls (*n* = 10) were sonicated to generate fragments between 200 and 1000 base pairs (bp), and then immunoprecipitated with a murine monoclonal antibody that specifically recognizes 5-methylcytidine (Diagenode, Liège, Belgium). The immunoprecipitated DNA fragments were recovered using anti-mouse IgG magnetic beads. The MeDIP-enriched DNA was amplified by PCR using universal primers and a limited number of cycles (GenomePlex® Complete Whole Genome Amplification kit). The amplified DNA samples were then purified with QIAquick PCR purification kit (Qiagen, Valencia, CA, USA). One microgram of immunoprecipitated and reference DNA was tagged respectively with Cyanine-5 (Cy5) and Cyanine-3 (Cy3) labeled random 9-mers and then hybridized by the NimbleGen Array Hybridization Kit (Roche NimbleGen, Madison, WI, USA). The labeled DNA was then purified by isopropanol/ethanol precipitation. For array hybridization, Roche NimbleGen’s Human Promoter plus CpG Island array was used, a 3 × 720k format array design containing 27,728 CpG islands and all well-characterized promoter regions (from about −2440 to +610 bp of the TSSs) totally covered by ~720,000 probes.

### Bisulfite sequencing

The CpG island DNA methylation status was determined by sequencing bisulfite-modified genomic DNA [43] from PBC patients (*n* = 20) and controls (*n* = 20). One microgram of genomic DNA was bisulfite-converted using the EpiTect Bisulfite Kit (Qiagen, Valencia, CA, USA). For each gene, primers were designed using the Methyl Primer Express v1.0 program (Life Technologies-Applied Biosystems, Carlsbad, CA, USA) corresponding to the region containing the oligonucleotide probe represented in the DNA methylation bead array. Ten positive recombinant clones were selected randomly and sequenced using ABI 3730 (Life Technologies-Applied Biosystems, Carlsbad, CA, USA). The methylation level of gene is assessed according to the percentage of the methylated CG sites in 10 clones. All primer sequences are listed in Additional file 1: Table S1.

### Reverse transcription and quantitative polymerase chain reaction

Total RNA was extracted from all isolated samples (PBC *n* = 30, controls *n* = 30) using TRI Reagent (Sigma-Aldrich, St. Louis, MO, USA). cDNA was synthesized from all different cell types using High Capacity cDNA Reverse Transcription Kits (Life Technologies-Applied Biosystems, Carlsbad, CA, USA). One hundred nanograms of cDNA in a total volume of 20 μl was amplified for 40 cycles on an Applied Biosystems 7900 HT Sequence Detection System, using a TaqMan Gene Expression Assay specific for CXCR3, UBE2A, FUNDC2, and IL1RAPL2 (Cat. #4331182, #4351372, Life Technologies-Applied Biosystems, Carlsbad, CA, USA). TaqMan® Gene Expression Assays consist of a pair of unlabeled PCR primers and a TaqMan® probe with a FAM™ dye label on the 5′ end and minor groove binder (MGB) nonfluorescent quencher (NFQ) on the 3′ end. RNA from samples of interest is reverse transcribed into cDNA, and the synthesized cDNA serves as the template for real-time PCR. ROX™ dye is used as a passive reference to normalize real-time PCR reactions. ROX™ dye provides an internal reference to which the FAM™ reporter dye signal can be normalized, which is necessary to correct for fluorescent fluctuations due to changes in concentration, volume, or light source intensity. Normalization of the reporter dye signal results in increased data precision. All reactions were run in duplicate. The relative mRNA expression level of each gene was determined by normalizing its mRNA level to the internal control glyceraldehyde 3-phosphate dehydrogenase (GAPDH).

### Flow cytometry

Phenotypic analysis of CD4 T cells was assessed using fluorochrome-conjugated monoclonal antibodies against cell surface markers on a FACSCantoII (BD Biosciences, San Diego, CA, USA) using FACSDiva™ software (BD Biosciences, San Diego, CA, USA). Monoclonal antibodies against human CD3, CD4, CD45RA, CD45RO, and CD183 (CXCR3) were purchased from BioLegend (San Diego, CA, USA). Frozen cells were thawed and prepared for flow cytometry as described [44]. Cells were stained for 15 min at room temperature with Aqua viability dye (Life Technologies). Optimal concentrations of the mAbs were used throughout, and all assays included positive and negative controls.

### Statistical methods

NimbleScan software v2.5 (Roche NimbleGen, Madison, WI, USA) was utilized for DNA methylation data analysis using a threshold *p* value of 0.05 equivalent to 1.31 based on the Gaussian distribution of data. Second, exclusive elements corresponding to specific microarray probes were identified using a one-sided Kolmogorov-Smirnov (KS) test in affected and healthy subjects; peaks found only in either group were selected for further analysis. Peaks within 500 bp of each other are merged. If several adjacent probes rise significantly above a set threshold, the region is assigned to an enrichment peak (EP). NimbleScan detects peaks by searching for at least two probes above a *p* value minimum cutoff (−log10) of 2. Third, elements of interest were inserted into the UCSC Genome Browser to identify corresponding genes. The filter condition of different methylated regions was set to be no less than six EPs in one group and not more than three EPs in the other group. Based on GO analysis and potential involvement in the pathogenesis of PBC, four genes were selected for validation using isolated CD4+, CD8+, and CD14+ cells from 20 additional patients with PBC and 20 additional normal controls. For RT-qPCR, the data were analyzed with Delta Delta Ct using global normalization. Statistical differences between groups were identified using an unpaired *t* test taking into account transcripts with *p* value <0.05.

## Additional file

Additional file 1:
**Supplementary table.**

## Abbreviations

AMAs: antimitochondrial antibodies
GAPDH: glyceraldehyde 3-phosphate dehydrogenase
GWAS: genome-wide association
IP10: IFNγ-inducible 10 kDa protein
KS: Kolmogorov-Smirnov
MeDIP: methylated DNA immunoprecipitation
PBC: primary biliary cirrhosis
RT-qPCR: reverse transcription and quantitative polymerase chain reaction

## References

[1] Sawalha AH (2008). Epigenetics and T-cell immunity. Autoimmunity.

[2] Coit P, Jeffries M, Altorok N, Dozmorov MG, Koelsch KA, Wren JD (2013). Genome-wide DNA methylation study suggests epigenetic accessibility and transcriptional poising of interferon-regulated genes in naive CD4+ T cells from lupus patients. J Autoimmun.

[3] Richardson BC, Patel DR (2014). Epigenetics in 2013: DNA methylation and miRNA—key roles in systemic autoimmunity. Nat Rev Rheumatol.

[4] Hewagama A, Gorelik G, Patel D, Liyanarachchi P, McCune WJ, Somers E (2013). Overexpression of X-linked genes in T cells from women with lupus. J Autoimmun.

[5] Shimoda S, Harada K, Niiro H, Shirabe K, Taketomi A, Maehara Y (2011). Interaction between Toll-like receptors and natural killer cells in the destruction of bile ducts in primary biliary cirrhosis. Hepatology.

[6] Lleo A, Bowlus CL, Yang GX, Invernizzi P, Podda M, Van de Water J (2010). Biliary apotopes and anti-mitochondrial antibodies activate innate immune responses in primary biliary cirrhosis. Hepatology.

[7] Invernizzi P (2014). Primary biliary cirrhosis. Semin Liver Dis.

[8] Kita H, Matsumura S, He XS, Ansari AA, Lian ZX, Van de Water J (2002). Quantitative and functional analysis of PDC-E2-specific autoreactive cytotoxic T lymphocytes in primary biliary cirrhosis. J Clin Invest.

[9] Kita H, Naidenko OV, Kronenberg M, Ansari AA, Rogers P, He XS (2002). Quantitation and phenotypic analysis of natural killer T cells in primary biliary cirrhosis using a human CD1d tetramer. Gastroenterology.

[10] Shimoda S, Van de Water J, Ansari A, Nakamura M, Ishibashi H, Coppel RL (1998). Identification and precursor frequency analysis of a common T cell epitope motif in mitochondrial autoantigens in primary biliary cirrhosis. J Clin Invest.

[11] Kaplan MM, Gershwin ME (2005). Primary biliary cirrhosis. N Engl J Med.

[12] Gershwin ME, Mackay IR (2008). The causes of primary biliary cirrhosis: convenient and inconvenient truths. Hepatology.

[13] Hirschfield GM, Liu X, Xu C, Lu Y, Xie G, Gu X (2009). Primary biliary cirrhosis associated with HLA, IL12A, and IL12RB2 variants. N Engl J Med.

[14] Liu X, Invernizzi P, Lu Y, Kosoy R, Bianchi I, Podda M (2010). Genome-wide meta-analyses identify three loci associated with primary biliary cirrhosis. Nat Genet.

[15] Mells GF, Floyd JA, Morley KI, Cordell HJ, Franklin CS, Shin SY (2011). Genome-wide association study identifies 12 new susceptibility loci for primary biliary cirrhosis. Nat Genet.

[16] Juran BD, Hirschfield GM, Invernizzi P, Atkinson EJ, Li Y, Xie G (2012). Immunochip analyses identify a novel risk locus for primary biliary cirrhosis at 13q14, multiple independent associations at four established risk loci and epistasis between 1p31 and 7q32 risk variants. Hum Mol Genet.

[17] Carbone M, Lleo A, Sandford RN, Invernizzi P (2014). Implications of genome-wide association studies in novel therapeutics in primary biliary cirrhosis. Eur J Immunol.

[18] Lleo A, Liao J, Invernizzi P, Zhao M, Bernuzzi F, Ma L (2012). Immunoglobulin M levels inversely correlate with CD40 ligand promoter methylation in patients with primary biliary cirrhosis. Hepatology.

[19] Mitchell MM, Lleo A, Zammataro L, Mayo MJ, Invernizzi P, Bach N (2011). Epigenetic investigation of variably X chromosome inactivated genes in monozygotic female twins discordant for primary biliary cirrhosis. Epigenetics: official J of the DNA Methylation Society.

[20] Bianchi I, Carbone M, Lleo A, Invernizzi P (2014). Genetics and epigenetics of primary biliary cirrhosis. Semin Liver Dis.

[21] Podda M, Selmi C, Lleo A, Moroni L, Invernizzi P (2013). The limitations and hidden gems of the epidemiology of primary biliary cirrhosis. J Autoimmun.

[22] Bianchi I, Lleo A, Gershwin ME, Invernizzi P (2012). The X chromosome and immune associated genes. J Autoimmun.

[23] Invernizzi P, Pasini S, Selmi C, Gershwin ME, Podda M (2009). Female predominance and X chromosome defects in autoimmune diseases. J Autoimmun.

[24] Invernizzi P, Miozzo M, Battezzati PM, Bianchi I, Grati FR, Simoni G (2004). Frequency of monosomy X in women with primary biliary cirrhosis. Lancet.

[25] Miozzo M, Selmi C, Gentilin B, Grati FR, Sirchia S, Oertelt S (2007). Preferential X chromosome loss but random inactivation characterize primary biliary cirrhosis. Hepatology.

[26] Lleo A, Oertelt-Prigione S, Bianchi I, Caliari L, Finelli P, Miozzo M (2013). Y chromosome loss in male patients with primary biliary cirrhosis. J Autoimmun.

[27] Chuang YH, Lian ZX, Cheng CM, Lan RY, Yang GX, Moritoki Y (2005). Increased levels of chemokine receptor CXCR3 and chemokines IP-10 and MIG in patients with primary biliary cirrhosis and their first degree relatives. J Autoimmun.

[28] Selmi C, Mayo MJ, Bach N, Ishibashi H, Invernizzi P, Gish RG (2004). Primary biliary cirrhosis in monozygotic and dizygotic twins: genetics, epigenetics, and environment. Gastroenterology.

[29] Richardson BC (2008). Epigenetics and autoimmunity. Overview. Autoimmunity.

[30] Javierre BM, Fernandez AF, Richter J, Al-Shahrour F, Martin-Subero JI, Rodriguez-Ubreva J (2010). Changes in the pattern of DNA methylation associate with twin discordance in systemic lupus erythematosus. Genome Res.

[31] Stefan M, Zhang W, Concepcion E, Yi Z, Tomer Y (2013). DNA methylation profiles in type 1 diabetes twins point to strong epigenetic effects on etiology. J Autoimmun.

[32] Shimoda S, Ishikawa F, Kamihira T, Komori A, Niiro H, Baba E (2006). Autoreactive T-cell responses in primary biliary cirrhosis are proinflammatory whereas those of controls are regulatory. Gastroenterology.

[33] Lohr H, Fleischer B, Gerken G, Yeaman SJ, Meyer zum Buschenfelde KH, Manns M (1993). Autoreactive liver-infiltrating T cells in primary biliary cirrhosis recognize inner mitochondrial epitopes and the pyruvate dehydrogenase complex. J Hepatol.

[34] Poupon R (2010). Primary biliary cirrhosis: a 2010 update. J Hepatol.

[35] Ramesh R, Kozhaya L, McKevitt K, Djuretic IM, Carlson TJ, Quintero MA (2014). Pro-inflammatory human Th17 cells selectively express P-glycoprotein and are refractory to glucocorticoids. J Exp Med.

[36] Duhen T, Campbell DJ (2014). IL-1beta promotes the differentiation of polyfunctional human CCR6+CXCR3+ Th1/17 cells that are specific for pathogenic and commensal microbes. J Immunol.

[37] O’Connor RA, Li X, Blumerman S, Anderton SM, Noelle RJ, Dalton DK (2012). Adjuvant immunotherapy of experimental autoimmune encephalomyelitis: immature myeloid cells expressing CXCL10 and CXCL16 attract CXCR3+CXCR6+ and myelin-specific T cells to the draining lymph nodes rather than the central nervous system. J Immunol.

[38] Oo YH, Weston CJ, Lalor PF, Curbishley SM, Withers DR, Reynolds GM (2010). Distinct roles for CCR4 and CXCR3 in the recruitment and positioning of regulatory T cells in the inflamed human liver. J Immunol.

[39] Antonelli A, Ferrari SM, Giuggioli D, Ferrannini E, Ferri C, Fallahi P (2014). Chemokine (C-X-C motif) ligand (CXCL)10 in autoimmune diseases. Autoimmun Rev.

[40] Zhang W, Fei Y, Gao J, Liu B, Zhang F (2011). The role of CXCR3 in the induction of primary biliary cirrhosis. Clin Dev Immunol.

[41] Groom JR, Richmond J, Murooka TT, Sorensen EW, Sung JH, Bankert K (2012). CXCR3 chemokine receptor-ligand interactions in the lymph node optimize CD4+ T helper 1 cell differentiation. Immunity.

[42] Chomczynski P, Mackey K (1995). Short technical reports. Modification of the TRI reagent procedure for isolation of RNA from polysaccharide- and proteoglycan-rich sources. Biotechniques.

[43] Fraga MF, Esteller M (2002). DNA methylation: a profile of methods and applications. Biotechniques.

[44] Lugli E, Gattinoni L, Roberto A, Mavilio D, Price DA, Restifo NP (2013). Identification, isolation and in vitro expansion of human and nonhuman primate T stem cell memory cells. Nat Protoc.

