# Supplementary material for: DNA methylation profiling of the X chromosome reveals an aberrant demethylation on CXCR3 promoter in primary biliary cirrhosis
Source: Clin Epigenetics. 2015 Jul 7;7(1):61. doi: 10.1186/s13148-015-0098-9 (PMC4491885; doi:10.1186/s13148-015-0098-9)
Supplement: Additional file 1: — Supplementary table. [file 13148_2015_98_MOESM1_ESM.doc]

Additional file 1: Table S1

| **Gene** | **Forward (5'-3')** | **Reverse (5'-3')** |
| --- | --- | --- |
| CXCR3 | AGTGTTGTTTTTGTTATAATTTATG | TACAAACAATAAAATTACCCTTCAC |
| UBE2A(outer primer) | TATTTTGGATTTTTTTGAATATAAA | ACTAAACACCACCCCAATATAAAC |
| UBE2A(internal primer) | ATTAAGGGGAAATGTGGTTTTAGA | AAATCCATCCCCACCTACTAATT |
| FUNDC2(outer primer) | TTATAGGTAAAATATTTTTTATTAGG | CAAAAAACTATTCCTAAATACCATCC |
| FUNDC2(internal primer) | GAGGGAGGGAGTTTTATTTT | ATAAAACAATTAAAAAAATACCTTATACAC |
| IL1RAPL2 | GATGGTTTATAGGGGAAGAGTGTATAA | CCAAATATCTTCCCAAAATATTTC |
